# Red culture immersion and psychological wellbeing among Chinese college students: the mediating role of collective self-esteem and the moderating role of psychological resilience

**DOI:** 10.3389/fpsyg.2026.1966132

**Published:** 2026-10-12

**Authors:** Yuting Wei

**Affiliations:** School of Humanities and Social Sciences, Yancheng Institute of Technology, Yancheng, Jiangsu, China

**Keywords:** collective self-esteem, college students, moderated mediation, psychological resilience, psychological wellbeing, red culture immersion

## Abstract

**Introduction:**

Red culture, the cultural legacy forged by the Chinese people under the leadership of the Communist Party of China during revolution, construction, and reform, is increasingly integrated into university education in China. However, the psychological mechanisms through which immersion in red culture contributes to college students’ psychological wellbeing remain largely unexplored. Drawing on social identity theory, the present study examined whether collective self-esteem mediates the relationship between red culture immersion and psychological wellbeing, and whether psychological resilience moderates these pathways.

**Methods:**

A cross-sectional survey was conducted with 612 Chinese college students (46.2% male; M age = 19.92, SD = 1.31) recruited from two universities in Jiangsu Province, China. Participants completed validated measures of red culture immersion, collective self-esteem, psychological resilience, and psychological wellbeing. A moderated mediation model was tested using structural equation modeling with latent interaction terms and bias-corrected bootstrap analyses (5,000 resamples).

**Results:**

Red culture immersion was positively associated with psychological wellbeing (*β* = 0.25, *p* < 0.001), and collective self-esteem partially mediated this relationship (indirect effect = 0.13, 95% CI [0.08, 0.18]). Psychological resilience moderated both the second stage of the mediation pathway and the direct pathway: the positive association between collective self-esteem and psychological wellbeing was stronger among students with high resilience (*β* = 0.43, *p* < 0.001) than among those with low resilience (*β* = 0.19, *p* < 0.001), and the direct effect of red culture immersion on psychological wellbeing was similarly amplified by resilience. The index of moderated mediation was significant (index = 0.10, 95% CI [0.05, 0.16]).

**Discussion:**

Immersion in red culture is associated with college students’ psychological wellbeing partly through stronger collective self-esteem, and this beneficial process is particularly pronounced among psychologically resilient students. These findings extend social identity theory to the domain of red culture education and carry practical implications for integrating red culture resources into university mental health education.

## 1 Introduction

College years constitute a critical developmental period in which young people consolidate their identities, values, and psychological resources, yet this period is also marked by elevated vulnerability to mental health difficulties (Keyes, 2002; Masten, 2001). In recent decades, the positive psychology movement has shifted scholarly attention from the mere absence of psychopathology toward the cultivation of flourishing, meaning, and optimal functioning (Seligman and Csikszentmihalyi, 2000), with psychological wellbeing defined as the realization of one’s true potential across multiple dimensions of positive functioning (Ryff, 1989). For Chinese college students, identifying contextual factors that nourish psychological wellbeing has become a priority for both educational research and mental health practice (Gao and McLellan, 2018).

Within the Chinese higher education context, red culture—the advanced culture created and accumulated by the Chinese people under the leadership of the Communist Party of China during the revolutionary, construction, and reform eras—has been systematically incorporated into university curricula and campus life as a core vehicle of ideological and moral education (Huang et al., 2023). Red culture resources, including revolutionary memorial sites, red music, patriotic films, and thematic educational activities, are widely used to cultivate students’ ideals, beliefs, and patriotic sentiments (He et al., 2025; Hu et al., 2024). Beyond its ideological value, educators have increasingly argued that red culture carries significant psychological functions, providing spiritual nourishment that may promote positive mental states and resilience in students (Huang et al., 2023). Nevertheless, rigorous empirical evidence on the psychological benefits of red culture immersion remains scarce, and the mechanisms linking such immersion to college students’ wellbeing are poorly understood.

A growing body of research indicates that cultural and historical engagement can promote psychological wellbeing through identity-related processes. For instance, a recent longitudinal study found that red musical identity positively predicted subjective wellbeing among Chinese university students through the chain mediation of awe and prosocial behavior (He et al., 2025), and civic education integrated into physical education courses was shown to enhance prosocial behavior through cultural confidence and self-esteem (Huang et al., 2023). These findings suggest that engagement with culturally meaningful content operates on wellbeing not merely as pleasant stimulation but as a source of identity affirmation. However, previous studies have focused on specific forms of red culture such as red music, leaving the general construct of red culture immersion—the cognitive, affective, and behavioral involvement of students in red culture learning and practice—largely unexamined.

Social identity theory offers a powerful lens for understanding why red culture immersion may benefit psychological wellbeing. According to this theory, a substantial part of the self-concept derives from membership in social groups, and the positive evaluation of one’s group memberships contributes to a positive social identity (Tajfel and Turner, 1979; Turner et al., 1987). The social identity approach to health proposes that group memberships function as psychological resources that provide meaning, support, and agency, thereby protecting and enhancing wellbeing (Haslam et al., 2009; Jetten et al., 2017). Red culture, as a collective cultural heritage shared by the nation, may serve as a particularly potent source of positive social identity: students who immerse themselves in red culture experience a heightened sense of belonging to a valued collective with a glorious history, which should be reflected in elevated collective self-esteem—the evaluative component of social identity (Luhtanen and Crocker, 1992). Consistent with this reasoning, a recent study of 583 Chinese college students demonstrated that national identity predicted subjective wellbeing through collective self-esteem (Zhao et al., 2026), and collective self-esteem has been shown to mediate the effects of cultural orientation on wellbeing across cultural groups (Yamaguchi et al., 2017).

The beneficial pathway from red culture immersion to wellbeing is unlikely to operate uniformly across individuals. Psychological resilience—the capacity to adapt positively and bounce back in the face of adversity (Connor and Davidson, 2003; Masten, 2001)—has been consistently identified as a protective factor that amplifies the positive effects of psychological resources on mental health (Cui et al., 2024; Wang et al., 2024). Resilient individuals are better able to harness positive experiences and translate psychological resources into wellbeing, suggesting that resilience may moderate both the direct effect of red culture immersion and the indirect effect via collective self-esteem. To date, however, no study has tested whether psychological resilience moderates the mediating pathway from red culture immersion to psychological wellbeing through collective self-esteem. Accordingly, the present study examined a moderated mediation model in which collective self-esteem mediates the relationship between red culture immersion and psychological wellbeing, and psychological resilience moderates the second stage of the mediation pathway as well as the direct pathway. We hypothesized that (H1) red culture immersion would positively predict psychological wellbeing; (H2) collective self-esteem would mediate the relationship between red culture immersion and psychological wellbeing; and (H3) psychological resilience would moderate the direct and indirect effects, such that the beneficial effects of red culture immersion and collective self-esteem would be stronger for students with higher resilience.

### 1.1 Social identity theory and red culture immersion

Social identity theory maintains that individuals define themselves partly in terms of the social groups to which they belong, and that the desire for a positive social identity motivates them to evaluate their groups favorably (Tajfel and Turner, 1979). Self-categorization theory further specifies that the cognitive process of categorizing the self as a group member transforms self-perception, aligning individual cognition, affect, and behavior with group norms and values (Turner et al., 1987). The social identity approach to health extends these insights to the domain of physical and mental health, proposing that group memberships provide meaning, social support, and a sense of agency, all of which constitute psychological resources that protect wellbeing (Haslam et al., 2009; Jetten et al., 2017).

Red culture immersion refers to the extent to which students engage with red culture across cognitive, affective, and behavioral domains—that is, how deeply they understand and reflect upon red cultural history and values, how strongly they resonate emotionally with revolutionary spirit, and how actively they participate in red culture learning and practice activities (He et al., 2025). Conceptually, immersion of this kind entails a state of deep involvement analogous to the flow experience described by Csikszentmihalyi (1990), in which the individual becomes absorbed in a meaningful activity. From a social identity perspective, red culture constitutes the collective heritage of the Chinese nation, and immersion in it activates a shared social category that connects the individual to a valued national community (He et al., 2025; Hu et al., 2024). Red culture immersion may therefore function as a context in which a positive social identity is constructed and maintained, with downstream benefits for psychological functioning.

### 1.2 Collective self-esteem as a mediator

Collective self-esteem refers to the positivity of individuals’ evaluations of the social groups to which they belong, encompassing membership esteem, private and public collective self-esteem, and the importance of social identity to the self (Luhtanen and Crocker, 1992; Crocker and Luhtanen, 1990). Whereas personal self-esteem concerns the evaluation of the individual self, collective self-esteem captures the evaluative dimension of social identity and is thus the psychological construct most directly implicated by social identity theory in the link between group engagement and wellbeing.

Theoretically, engagement with a valued cultural collective should enhance collective self-esteem because it reinforces the perception that one’s group is worthy, positively regarded, and central to the self (Crocker and Luhtanen, 1990). Red culture immersion, by connecting students to a heroic national history and a community of shared values, should strengthen the sense that one belongs to a valuable collective, thereby elevating collective self-esteem. In turn, collective self-esteem is a documented correlate of wellbeing. Yamaguchi et al. (2017) found that collective self-esteem mediated the effects of cultural orientation on subjective wellbeing. More directly relevant, Zhao et al. (2026) demonstrated in a sample of Chinese college students that national identity predicted subjective wellbeing through collective self-esteem, and Herdian et al. (2025) reported that collective self-esteem, particularly its public dimension, mediated the association between ethnic identity and mental health among university students. These findings converge on the hypothesis that collective self-esteem is a key mechanism translating cultural identification into psychological wellbeing, and we accordingly expected it to mediate the red culture immersion–wellbeing relationship.

### 1.3 Psychological wellbeing

Psychological wellbeing, as conceptualized by Ryff (1989), is a multidimensional construct encompassing self-acceptance, positive relations with others, autonomy, environmental mastery, purpose in life, and personal growth, reflecting eudaimonic rather than merely hedonic functioning. This conceptualization contrasts with, yet complements, subjective wellbeing, which emphasizes life satisfaction and the balance of positive and negative affect (Diener, 1984; Diener et al., 2018). The eudaimonic perspective is particularly relevant to the study of red culture, whose educational purpose is precisely to cultivate purpose, values, and growth-oriented meaning in young people (Huang et al., 2023). The Ryff scales have been validated for use with Chinese adolescents and young adults (Gao and McLellan, 2018), supporting their applicability in the present context. In this study, psychological wellbeing was operationalized as the six-dimensional eudaimonic functioning described by Ryff (1989) and Ryff and Keyes (1995).

Self-determination theory offers a complementary motivational account (Ryan and Deci, 2000). Immersive participation in collectively valued cultural activities can satisfy the basic psychological need for relatedness, because shared engagement with a common heritage connects the self to a valued community; sustained need satisfaction of this kind is precisely what self-determination theory links to eudaimonic rather than merely hedonic functioning. Red culture immersion may therefore feed psychological wellbeing through a motivational route that complements the identity-based pathway developed above.

### 1.4 Psychological resilience as a moderator

Psychological resilience is defined as the ability to adapt positively to stress, adversity, and challenge, and to bounce back from difficult experiences (Connor and Davidson, 2003; Masten, 2001). The Connor-Davidson Resilience Scale (CD-RISC) and its Chinese version have been widely used to assess resilience as a trait-like capacity comprising tenacity, strength, and optimism (Connor and Davidson, 2003; Yu and Zhang, 2007). Resilience is not merely the absence of distress but an active psychological resource that shapes how individuals process and capitalize on life experiences (Smith et al., 2008).

Evidence from Chinese university students indicates that resilience amplifies the beneficial effects of positive psychological resources on mental health. Cui et al. (2024) found that resilience mediated and moderated the relationships between self-esteem, personality traits, and mental wellbeing among 689 Chinese university students, such that resilient students were better able to translate self-esteem into wellbeing. Similarly, Wang et al. (2024) showed that resilience helped stabilize emotions and buffer the psychological consequences of stressful environments. These findings suggest that resilient individuals are more likely to convert identity-enhancing experiences, such as red culture immersion and the collective self-esteem it cultivates, into tangible gains in psychological wellbeing. We therefore expected resilience to moderate both the direct effect of red culture immersion on wellbeing and the second-stage effect of collective self-esteem on wellbeing.

### 1.5 The present study

In summary, the existing literature establishes (a) that red culture education is a distinctive and valued feature of Chinese higher education (He et al., 2025; Huang et al., 2023), (b) that social identity processes, especially collective self-esteem, translate group and cultural engagement into wellbeing (Haslam et al., 2009; Zhao et al., 2026), and (c) that resilience moderates the translation of psychological resources into mental health (Cui et al., 2024; Wang et al., 2024). However, no study has integrated these strands to examine whether collective self-esteem mediates the relationship between red culture immersion and psychological wellbeing, and whether resilience moderates this process. The broaden-and-build theory further suggests that positive emotional experiences, such as those evoked by meaningful cultural engagement, broaden cognition and build enduring psychological resources, with resilient individuals profiting most from such experiences (Fredrickson, 2001). Accordingly, we tested a moderated mediation model with three hypotheses: red culture immersion positively predicts psychological wellbeing (H1); collective self-esteem mediates the red culture immersion–psychological wellbeing relationship (H2); and psychological resilience moderates the direct and mediated pathways such that their effects are stronger at higher levels of resilience (H3).

## 2 Method

### 2.1 Participants and procedure

Participants were 612 undergraduate students recruited from two universities in Jiangsu Province, China, during the spring semester of 2025. Of these, 283 (46.2%) were male and 329 (53.8%) were female, with ages ranging from 17 to 25 years (*M* = 19.92, SD = 1.31). Students came from a range of majors, including humanities and social sciences, engineering, and education. All participants had taken part in red culture education activities organized by their universities (e.g., visits to revolutionary memorial sites, red-themed courses, and patriotic film screenings).

Data were collected through an anonymous paper-and-pencil survey administered in classroom settings by trained research assistants. Participants were informed of the purpose of the study, the voluntary nature of their participation, and the confidentiality of their responses, and they provided written informed consent before completing the questionnaire. The survey took approximately 15–20 min to complete. The study was approved by the Institutional Review Board of Yancheng Institute of Technology and was conducted in accordance with the principles of the Declaration of Helsinki.

### 2.2 Measures

#### 2.2.1 Red culture immersion

Red culture immersion was measured with a 12-item scale developed for this study. Drawing on the tripartite conceptualization of red cultural identity as comprising cognitive-emotional, cultural, and behavioral dimensions (He et al., 2025) and on the notion of immersive engagement with meaningful activities (Csikszentmihalyi, 1990), the scale assesses three facets of red culture immersion: cognitive immersion (e.g., “I often reflect on the historical significance of red culture”), affective immersion (e.g., “I feel deeply moved when I learn about the revolutionary spirit”), and behavioral immersion (e.g., “I actively participate in red culture learning activities organized by my university”). Items were rated on a 5-point Likert scale ranging from 1 (strongly disagree) to 5 (strongly agree). The content validity of the scale was evaluated by two professors in educational psychology, and a pilot study with 80 students confirmed adequate internal consistency. In the present sample, the scale demonstrated good reliability (*α* = 0.89), and a confirmatory factor analysis supported the three-factor structure, *χ*^2^(51) = 108.42, CFI = 0.96, RMSEA = 0.043.

#### 2.2.2 Collective self-esteem

Collective self-esteem was measured with the 16-item Collective Self-Esteem Scale developed by Luhtanen and Crocker (1992). The scale comprises four subscales: membership esteem, private collective self-esteem, public collective self-esteem, and importance to identity. Items (e.g., “I feel I am a worthy member of the social groups I belong to”) were rated on a 7-point Likert scale ranging from 1 (strongly disagree) to 7 (strongly agree), with negatively worded items reverse-scored. The Chinese version of the scale has been used with Chinese college students in prior research (Zhao et al., 2026). In the present sample, Cronbach’s α was 0.87.

#### 2.2.3 Psychological resilience

Psychological resilience was assessed with the Chinese version of the 25-item Connor-Davidson Resilience Scale (CD-RISC; Connor and Davidson, 2003), which was translated and validated for Chinese populations by Yu and Zhang (2007). The scale comprises three dimensions: tenacity, strength, and optimism. Items (e.g., “I am able to adapt when changes occur”) were rated on a 5-point Likert scale ranging from 0 (not true at all) to 4 (true nearly all of the time). The Chinese CD-RISC has demonstrated sound psychometric properties in Chinese samples (Yu and Zhang, 2007; Cui et al., 2024). In the present sample, Cronbach’s *α* was 0.91.

#### 2.2.4 Psychological wellbeing

Psychological wellbeing was measured with the 18-item short form of the Ryff Psychological WellBeing Scales (Ryff, 1989; Ryff and Keyes, 1995), which assesses the six dimensions of self-acceptance, positive relations with others, autonomy, environmental mastery, purpose in life, and personal growth, with three items per dimension. Items (e.g., “I have confidence in my own opinions, even if they are contrary to the general consensus”) were rated on a 6-point Likert scale ranging from 1 (strongly disagree) to 6 (strongly agree). The Ryff scales have been validated for use with adolescents and young adults in mainland China (Gao and McLellan, 2018). In the present sample, Cronbach’s α for the total scale was 0.89.

### 2.3 Data analysis

Descriptive statistics and Pearson correlations were computed with SPSS 26.0. Confirmatory factor analysis (CFA) and structural equation modeling (SEM) were conducted with Mplus 8.3 (Muthén and Muthén, 2017). Model fit was evaluated using the chi-square statistic, the comparative fit index (CFI), the Tucker-Lewis index (TLI), the root mean square error of approximation (RMSEA), and the standardized root mean square residual (SRMR), with CFI and TLI ≥ 0.90 and RMSEA and SRMR ≤ 0.08 indicating acceptable fit (Hu and Bentler, 1999). To test the moderated mediation model, latent interaction terms were constructed using the latent moderated structural equations (LMS) approach (Klein and Moosbrugger, 2000). Bias-corrected bootstrap confidence intervals based on 5,000 resamples were used to test the indirect effects and the index of moderated mediation (Preacher et al., 2007; Hayes, 2015). Simple slopes for the moderator were examined at one standard deviation below and above the mean, following the recommendations of Hayes (2018). Given the absence of multivariate normality, all bootstrap confidence intervals were computed with the MLR estimator (maximum likelihood estimation with standard errors adjusted for non-normality).

## 3 Results

### 3.1 Descriptive statistics and correlations

Table 1 presents the descriptive statistics, reliability coefficients, and bivariate correlations among the study variables. All variables approximated a normal distribution (skewness < |1.0|; kurtosis < |1.5|). On average, participants reported moderate levels of red culture immersion (*M* = 3.42, SD = 0.71), collective self-esteem (*M* = 4.76, SD = 0.89), psychological resilience (*M* = 2.96, SD = 0.62), and psychological wellbeing (*M* = 4.38, SD = 0.77). As expected, red culture immersion was positively correlated with collective self-esteem (*r* = 0.41, *p* < 0.001), psychological wellbeing (*r* = 0.45, *p* < 0.001), and psychological resilience (*r* = 0.28, *p* < 0.001). Collective self-esteem was positively correlated with psychological wellbeing (*r* = 0.52, *p* < 0.001) and psychological resilience (*r* = 0.34, *p* < 0.001), and psychological resilience was positively correlated with psychological wellbeing (*r* = 0.39, *p* < 0.001).

**Table 1:** Descriptive statistics, reliability coefficients, and bivariate correlations among study variables (*N* = 612).

Variable	*M*	SD	*α*	1	2	3	4
1. Red culture immersion	3.42	0.71	0.89	–			
2. Collective self-esteem	4.76	0.89	0.87	0.41^***^	–		
3. Psychological resilience	2.96	0.62	0.91	0.28^***^	0.34^***^	–	
4. Psychological wellbeing	4.38	0.77	0.89	0.45^***^	0.52^***^	0.39^***^	–

*M* = mean; SD = standard deviation; *α* = Cronbach’s alpha. ^***^p < 0.001.

### 3.2 Measurement model

Confirmatory factor analysis was conducted to examine the factor structure of the four latent variables. The four-factor model provided a good fit to the data, *χ*^2^(237) = 498.32, CFI = 0.95, TLI = 0.94, RMSEA = 0.043 (90% CI [0.037, 0.048]), SRMR = 0.049. All standardized factor loadings were significant and ranged from 0.52 to 0.88, indicating that the latent variables were adequately measured.

### 3.3 Structural model with latent interaction

To test the moderated mediation model, latent interaction terms (collective self-esteem × psychological resilience and red culture immersion × psychological resilience) were created using the latent moderated structural equations (LMS) approach (Klein and Moosbrugger, 2000). The model including the interaction terms fit the data well, *χ*^2^(365) = 789.54, CFI = 0.93, TLI = 0.92, RMSEA = 0.045 (90% CI [0.040, 0.050]), SRMR = 0.055. As shown in Table 2, red culture immersion was positively associated with both collective self-esteem (*β* = 0.41, *p* < 0.001) and psychological wellbeing (*β* = 0.25, *p* < 0.001), supporting H1. Collective self-esteem was positively associated with psychological wellbeing (*β* = 0.31, *p* < 0.001). Importantly, the collective self-esteem × resilience interaction (*β* = 0.12, *p* = 0.004) and the red culture immersion × resilience interaction (*β* = 0.08, *p* = 0.031) were both significantly associated with psychological wellbeing. The model explained 17% of the variance in collective self-esteem and 44% of the variance in psychological wellbeing. The full structural model with standardized path estimates is depicted in Figure 1.

**Table 2:** Standardized path estimates, conditional indirect effects, and index of moderated mediation.

Path	*β*	SE	95% CI	*p*
Red culture immersion → Collective self-esteem	0.41	0.04	[0.33, 0.49]	<0.001
Collective self-esteem → Psychological wellbeing	0.31	0.05	[0.22, 0.40]	<0.001
Red culture immersion → Psychological wellbeing	0.25	0.05	[0.15, 0.35]	<0.001
Collective self-esteem × Resilience → Wellbeing	0.12	0.04	[0.04, 0.20]	0.004
Red culture immersion × Resilience → Wellbeing	0.08	0.04	[0.01, 0.16]	0.031
Indirect: RCI → CSE → PWB (total)	0.13	0.03	[0.08, 0.18]	sig.
Conditional indirect: Low resilience (−1 SD)	0.08	0.02	[0.04, 0.13]	sig.
Conditional indirect: Moderate resilience	0.13	0.03	[0.08, 0.18]	sig.
Conditional indirect: High resilience (+1 SD)	0.18	0.03	[0.12, 0.25]	sig.
Index of moderated mediation	0.10	0.03	[0.05, 0.16]	sig.

*β* = standardized estimate; SE = standard error; CI = bias-corrected confidence interval based on 5,000 bootstrap resamples. RCI = red culture immersion; CSE = collective self-esteem; PWB = psychological wellbeing. “sig.” = confidence interval excludes zero. Conditional effects were estimated at one standard deviation below and above the mean of psychological resilience.

**Figure 1:**
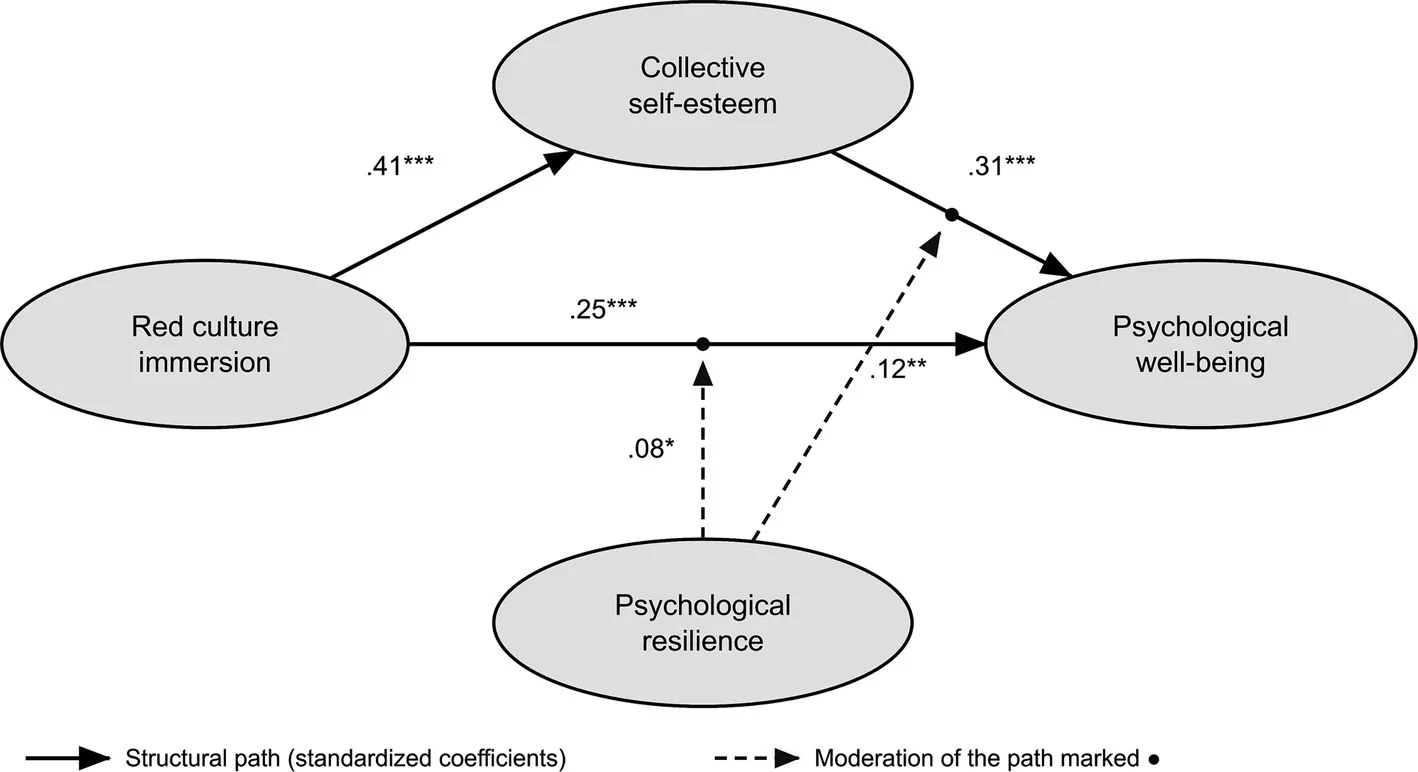
Structural equation model depicting the moderated mediation among red culture immersion, collective self-esteem, psychological resilience, and psychological wellbeing, with standardized path estimates (*N* = 612). *p <.05, **p <.01, ***p <.001.

### 3.4 Mediation and moderated mediation

Bias-corrected bootstrap analyses (5,000 resamples) were used to test the indirect effect of red culture immersion on psychological wellbeing through collective self-esteem (Preacher et al., 2007; Hayes, 2015). The total indirect effect was significant (*β* = 0.13, 95% CI [0.08, 0.18]), supporting H2. Conditional indirect effects at different levels of psychological resilience are reported in Table 2. The indirect effect was significant at low (−1 SD) levels of resilience (*β* = 0.08, 95% CI [0.04, 0.13]), at moderate levels (*β* = 0.13, 95% CI [0.08, 0.18]), and at high (+1 SD) levels (*β* = 0.18, 95% CI [0.12, 0.25]). The index of moderated mediation was significant (index = 0.10, 95% CI [0.05, 0.16]), indicating that the indirect effect increased as psychological resilience increased, supporting H3.

### 3.5 Simple slope analyses

Simple slope analyses were conducted to probe the significant interactions (Hayes, 2018). For the collective self-esteem → psychological wellbeing path, the slope was significant at both low resilience (*b* = 0.19, *p* < 0.001) and high resilience (*b* = 0.43, *p* < 0.001), and the association was stronger at high resilience. For the direct path from red culture immersion to psychological wellbeing, the slope was significant at both low resilience (*b* = 0.17, *p* = 0.008) and high resilience (*b* = 0.33, *p* < 0.001). These results indicate that the beneficial effects of red culture immersion and collective self-esteem on psychological wellbeing are both amplified among psychologically resilient students.

## 4 Discussion

### 4.1 The direct effect of red culture immersion

Consistent with H1 and with recent evidence that engagement with red culture promotes wellbeing (He et al., 2025; Huang et al., 2023), red culture immersion was positively associated with psychological wellbeing even after accounting for collective self-esteem. This finding adds to the small but growing literature documenting the psychological benefits of red culture education in Chinese higher education (Hu et al., 2024) and provides quantitative support for the claim that red culture functions not only as an ideological resource but also as a psychological resource for college students.

### 4.2 The mediating role of collective self-esteem

Consistent with H2, collective self-esteem partially mediated the relationship between red culture immersion and psychological wellbeing. Students who immersed themselves more deeply in red culture reported higher collective self-esteem, which in turn was associated with greater psychological wellbeing. This pattern aligns with social identity theory, which holds that positive evaluations of one’s group memberships are a core source of a positive social identity and psychological wellbeing (Tajfel and Turner, 1979; Haslam et al., 2009; Jetten et al., 2017), and converges with recent empirical evidence that collective self-esteem transmits the effects of cultural and national identification to wellbeing among Chinese college students (Zhao et al., 2026; Yamaguchi et al., 2017; Herdian et al., 2025). Whereas personal self-esteem concerns the evaluation of the individual self (Rosenberg, 1965), collective self-esteem captures the evaluative dimension of social identity, and our findings suggest that red culture immersion contributes to wellbeing precisely by strengthening this collective dimension of the self.

### 4.3 The moderating role of psychological resilience

Consistent with H3, psychological resilience moderated both the second stage of the mediation pathway and the direct pathway. The positive associations of collective self-esteem and red culture immersion with psychological wellbeing were stronger among students with high resilience than among those with low resilience. These results echo previous findings that resilience amplifies the translation of psychological resources into mental health among Chinese university students (Cui et al., 2024; Wang et al., 2024) and extend them by demonstrating that resilience also strengthens the identity-based pathway from cultural engagement to wellbeing. Resilient students appear better able to capitalize on the identity-enhancing and meaning-providing experiences afforded by red culture immersion.

### 4.4 Theoretical contributions

The present findings make several theoretical contributions. First, we extend social identity theory to the domain of red culture education, demonstrating that the identity-based pathway from collective engagement to wellbeing—well established in intergroup and organizational contexts (Haslam et al., 2009; Jetten et al., 2017)—operates in the context of national cultural heritage education in China. Second, the moderated mediation pattern is consistent with the broaden-and-build theory (Fredrickson, 2001): meaningful cultural immersion may broaden cognition and build psychological resources, and resilient individuals are best positioned to profit from such experiences. Third, from a self-determination theory perspective (Ryan and Deci, 2000), red culture immersion may satisfy fundamental needs for relatedness and meaning, thereby supporting eudaimonic wellbeing (Ryff, 1989).

### 4.5 Practical implications

Practically, our findings suggest that red culture resources can be integrated into university mental health education as a means of promoting students’ psychological wellbeing. Universities should design red culture education activities that promote deep cognitive, affective, and behavioral immersion rather than passive exposure, so as to strengthen collective self-esteem. At the same time, because the benefits of red culture immersion are amplified among resilient students, universities should also invest in resilience-building programs—for example, through counseling services, resilience training, and supportive peer networks—so that students with lower resilience can also benefit from red culture education.

### 4.6 Limitations and future directions

Several limitations should be acknowledged. First, the cross-sectional design precludes causal inference, and the reverse direction cannot be excluded: students with higher pre-existing psychological wellbeing may be more inclined to immerse themselves in red culture activities. A longitudinal study tracking students from their first to their final year, or a quasi-experimental study comparing a cohort receiving a new immersive red culture curriculum with a matched control cohort, would be necessary to establish the temporal order and causal status of the observed associations. Second, all constructs were assessed with self-report measures collected at a single time point, which may be subject to common method bias and social desirability responding. Procedural remedies were applied during data collection (anonymity, voluntary participation, and confidentiality assurances), and Harman’s single-factor test showed that the first unrotated factor accounted for 18.8% of the total variance, which did not indicate serious common method bias (Podsakoff et al., 2003); nevertheless, statistical controls such as marker variables were not included, and residual bias cannot be ruled out. To create a richer picture, future studies could adopt a mixed-methods approach that combines surveys with in-depth interviews, behavioral observation, or experience sampling of students’ engagement in red culture activities. Third, the analyses did not control for potential confounding variables (such as family support, peer relationships, financial pressure, academic stress, personality traits, and prior mental-health difficulties) that may also shape psychological wellbeing; the estimated mediation and moderation effects may therefore be somewhat overestimated, and future studies should incorporate such covariates. Fourth, the sample was recruited through convenience sampling from only two universities in a single province (Jiangsu), which limits the generalizability of the findings to other regions, institutional types, and student populations. Fifth, future research could incorporate explicit measures of cultural and national identity (Phinney and Ong, 2007), examine additional mediators such as awe and prosocial behavior (He et al., 2025), and test whether the observed pattern generalizes across different forms of red culture (e.g., red tourism, red music, revolutionary literature).

## 5 Conclusion

The present study demonstrates that red culture immersion is positively associated with psychological wellbeing among Chinese college students, that collective self-esteem partially mediates this relationship, and that psychological resilience moderates both the direct and mediated pathways. These findings extend social identity theory to the domain of red culture education and highlight the psychological value of red culture as a resource for college students’ wellbeing. For educational practice, integrating red culture education with mental health promotion—and building students’ psychological resilience alongside their cultural identity—may offer a promising pathway to supporting the flourishing of college students.

## Data Availability

The raw data supporting the conclusions of this article will be made available by the authors, without undue reservation.
